# *Neisseria meningitidis* Serogroup X in Sub-Saharan Africa

**DOI:** 10.3201/eid2204.150653

**Published:** 2016-04

**Authors:** Alain Agnememel, Eva Hong, Dario Giorgini, Viginia Nuñez-Samudio, Ala-Eddine Deghmane, Muhamed-Kheir Taha

**Affiliations:** Institut Pasteur, Paris, France

**Keywords:** meningitis, sub-Saharan Africa, meningococci, whole-genome sequencing, virulence, emergence, bacteria, *Neisseria meningitidis*, serogroup X

## Abstract

The epidemiology of meningococcal disease varies by geography and time. Whole-genome sequencing of *Neisseria meningitidis* serogroup X isolates from sub-Saharan Africa and Europe showed that serogroup X emergence in sub-Saharan Africa resulted from expansion of particular variants within clonal complex 181. Virulence of these isolates in experimental mouse models was high.

The epidemiology of meningococcal disease, caused by infection with the bacterium *Neisseria meningitidis*, varies substantially by geography and time. The disease can occur as sporadic cases, outbreaks, and large epidemics. Most cases occur in what has been termed the “meningitis belt” in sub-Saharan Africa, where the World Health Organization estimated that 12,464 suspected cases occurred during the 2013 meningitis season and that 1,131 were fatal (case-fatality ratio 9%) (*1*).

The number of cases was lower during the 2013 season than during previous seasons and can be explained by the introduction of mass vaccination with the meningococcal A conjugate vaccine MenAfriVac (Serum Institute of India, Pune, India) (*1*). Cyclic epidemics spanned the meningitis belt and were caused mainly by isolates of *N. meningitidis* serogroup A that belonged to several genetic lineages called clonal complexes (CCs). Molecular typing of meningococci has used multilocus sequence typing (MLST) to determine CCs on the basis of the polymorphism of 7 housekeeping genes (*2*). Since the 1990s, isolates of other *N. meningitidis* serogroups, such as serogroup X, have been reported in several countries within the meningitis belt (*3*). In 2006, high incidence of serogroup X was first reported in Niamey, Niger (*4*), and thereafter in other countries, reinforcing the need for an effective vaccine against serogroup X (*5*). Serogroup X has rarely been detected in Europe (*6*). 

MLST may not be able to resolve the variations associated with the emergence of meningococcal isolates. Whole-genome sequencing can provide data that enable allelic comparisons between meningococcal isolates (*7*,*8*) and tracing of the emergence and spread of meningococcal isolates.

## The Study

As part of our mission as a World Health Organization collaborating center, we performed MLST on *N. meningitidis* serogroup X isolates sent to the Institut Pasteur during 1998–2005 (Table 1). We further performed whole-genome sequencing on 8 invasive serogroup X isolates obtained during 1998–2005 from several countries within the meningitis belt and 3 invasive isolates from France (Table 1).

**Table 1:** Characteristics of *Neisseria meningitidis* serogroup X isolates, sub-Saharan Africa and France, 1995–2008*

Isolate	Year collected	Country	Clonal complex	Allele *lpt3*	Reactivity of monoclonal antibody immunotype L3,7, 9
LNP13407	1995	Chad	ST-181	45	–
LNP14354	1996	Niger	ST-181	136	+
LNP14355	1996	Niger	ST-181	136	+
LNP15075	1997	Burkina Faso	ST-181	136	+
LNP14964	1997	Niger	ST-181	136	+
LNP19504	2002	France	ST-254	1	+
LNP23557	2006	Niger	ST-181	45	–
LNP2006100	2006	Niger	ST-181	45	–
LNP23552	2006	Niger	ST-181	45	–
2005166	2006	Niger	ST-181	45	–
LNP23558	2006	Niger	ST-181	45	–
LNP24287	2007	France	ST-750	–	+
LNP24196	2007	France	Unassigned	–	+
2008223B	2008	Burkina Faso	ST-181	45	–
2008112	2008	Benin	ST-181	45	–

*ST, sequence type; –, absent; +, present.

Genomic DNA was extracted by using a Genomic-tip 20/G kit (QIAGEN, Valencia CA, USA) from culture grown overnight on gonococcal medium base agar plates with Kellogg supplements (*9*). Whole-genome sequencing was performed by using an Illumina HiSeq 2000 sequencer (Illumina, San Diego, CA, USA) and assembled as described (*10*). Sequences are available through the PubMLST database (http://pubmlst.org/neisseria/), which runs on the Bacterial Isolate Genome Sequence Database (BIGSdb) platform (*11*); identification numbers are 34731–34732, 34734–34741, and 34745. We analyzed the 11 isolates from sub-Saharan Africa and France and all other serogroup X genomes available in BIGSdb by using the BIGSdb genome comparator tool.

**Figure 1:**
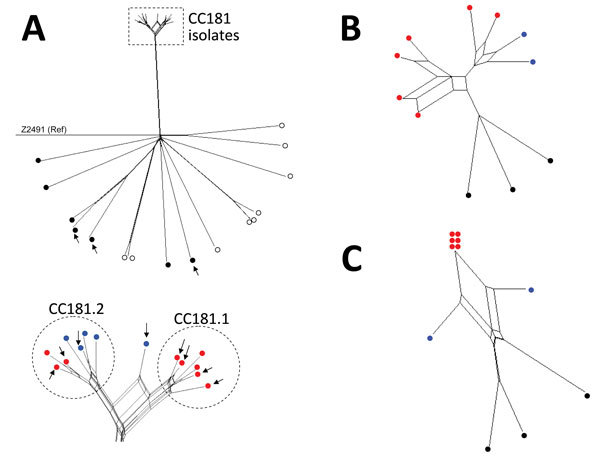
Neighbor-Net SplitsTree graphs generated using SplitsTree4 version 4.13.1 (http://www.splitstree.org) to visualize trees of *Neisseria meningitidis* serogroup X isolates. A) All 30 *Neisseria meningitidis* serogroup X isolates available on BIGSdb (*11*) were analyzed, including the 11 isolates from this study (8 from sub-Saharan Africa and 3 from France), 9 carriage isolates, 3 invasive isolates from Europe, 1 isolate from the United States, and 6 isolates from sub-Saharan African countries. Open (white) circles indicate carriage isolates; black circles indicate invasive isolates obtained outside of Africa; red circles indicate isolates obtained in sub-Saharan Africa since 2006; blue circles indicate isolates obtained from sub-Saharan Africa during the 1990s. Arrows indicate the 11 isolates obtained during this study. All isolates were compared with reference (Ref) meningococcal strain Z2491. The dashed rectangle indicates the cluster of all the clonal complex (CC) 181 isolates from sub-Saharan Africa (enlarged view at bottom of panel). B) The 11 isolates obtained during this study were compared for iron-acquisition genes (*tpbA*, *tbpB*, *hpuA*, *hpuB*, *lbpA*, *lbpB*, and *hmbR*). C) Genes of the 11 isolates obtained during this study compared with the 41 genes that differed between all the isolates obtained in Africa since 2006 and the isolate LNP13407 or the isolate LNP14354 (obtained during the 1990s).

The isolates from Africa were genetically related, belonged to CC181, and were separated from all the other isolates that belonged to other CCs (Table 1; Figure 1, panel A). The CC181 isolates formed a single main lineage comprising 2 sublineages, 181.1 and 182.2. Sublineage 181.1 was detected only in isolates obtained in 2006, but sublineage 181.2 was detected in isolates from both periods (1990s and since 2006). Genomes from the 3 isolates from France and those available in the PubMLST database were highly diverse with no close clustering (Figure 1, panel A).

Because we assembled the sequences by using the same method, we further focused on the 11 genomes according to particular groups of genes involved in meningococcal virulence (biosynthesis of the capsule, serogroup B vaccine antigens, lipooligosaccharide, pilin, and iron acquisition). Among genes involved in iron acquisition, the isolates from Africa, unlike those from France, lacked the *hpuA* and *hpuB* genes that mediate heme-iron acquisition from hemoglobin and hemoglobin–haptoglobin complexes. However, the isolates from Africa harbored the hemoglobin receptor that was missing in the isolates from France. The hemoglobin receptor is detected more frequently among isolates involved with disease than among those involved with carriage (*12*). The analysis of the polymorphism of these iron-acquisition genes showed that the isolates from Africa clustered together, separate from the isolates from France (Figure 1, panel B).

**Figure 2:**
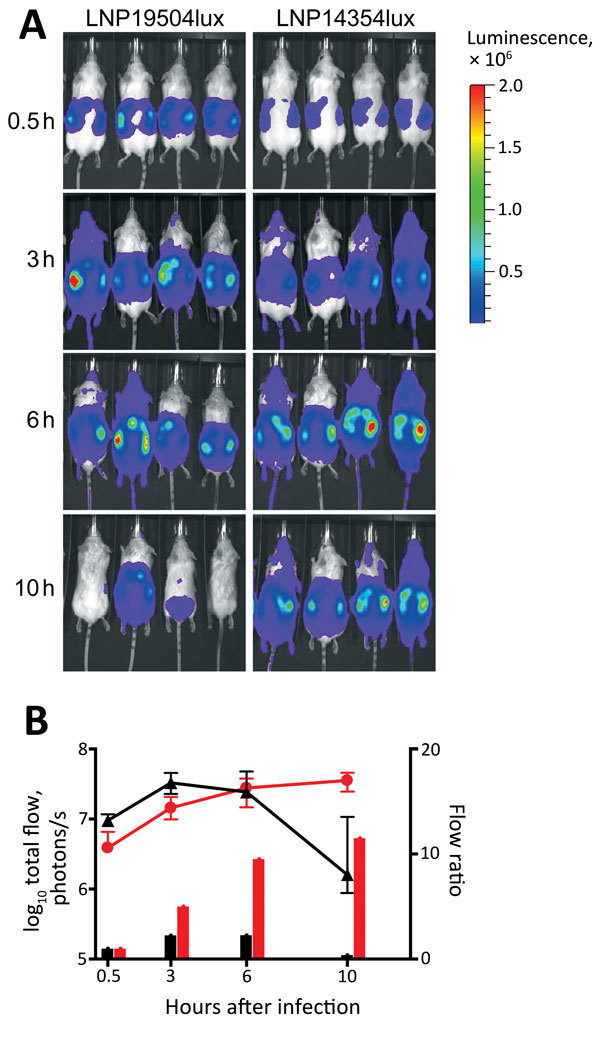
Dynamic imaging showing the multiplication and spread of *Neisseria meningitidis* in BALB/c transgenic mice expressing the human transferrin. A) Dorsal views of 8 mice (4/group) analyzed for bioluminescence at intervals after infection, as shown on left. Mice were infected by intraperitoneal injection of 5 × 10^6^ CFU of *N. meningitidis* strain LNP19504lux (derived from an isolate from France) or LNP14354lux (derived from an isolate from Africa). Both strains expressed the luciferase (*lux*) operon. Photographs are overlaid with color representations of luminescence intensity, measured in total photons per second and indicated on the scales: red, most intense (2.00 × 10^6^ p/s/cm^2^/steradian); blue, least intense (1.00 × 10^4^ p/s/cm^2^/steradian). B) Luminescence for LNP19504lux (black) and LNP14354lux (red) was quantified and expressed by using GraphPad PRISM version 4.00 (http://www.graphpad.com/). Means ± 95% CIs of total photons per second (lines, left y-axis) were calculated by defining the specific representative region of interest encompassing the entire animal. Signals differed significantly between the 2 groups after 10 h of infection (p = 0.01 by *t*-test). After 24 h, all mice had survived and signals declined for both isolates but remained detectable for LNP14354lux (not shown). The data are also expressed as flow ratio (total photons per second for each point/total photons at the first point [after 0.5 h of infection]) (bars, right y-axis).

We tested the virulence of these isolates in a relevant animal model, transgenic mice expressing the human transferrin (*13*). The animal experiments were conducted in accordance with the European Union Directive 2010/63/EU (and its revision 86/609/EEC) for the protection of animals used for scientific purposes and were approved by the Institut Pasteur Review Board, which is part of the Regional Committee of Ethics of Animal Experiments of the Paris region (permit 99–174). We constructed bioluminescent variants of isolates LNP19504 and LNP14354 by transformation with the previously published recombinant plasmid pDG34 harboring the *luxCDABE* operon expressed by the PporB meningococcal promoter (*14*). The structure was recombined in *N. meningitidis* downstream of the recombinogenic *pilE* gene to obtain the strains LNP19504lux and LNP14354lux. The insertion of the *lux* operon did not modify bacterial invasion in this animal model (*13*). These 2 bioluminescent strains were then used to infect transgenic mice by intraperitoneal injection of 5 × 10^6^ CFU in 500 μL of the corresponding bacterial suspension. Dynamic bioluminescence imaging was used to follow the infection. After 30 minutes of intraperitoneal challenge, bacteria were found mainly in the peritoneal cavity and the image signals did not differ substantially. Bacterial infection then started to spread, enabling detection of bioluminescent signals at distal anatomic sites; signals in the skull and all over the infected mice suggested systemic infection and septicemia (*13*). However, after 6 hours of infection, the signal decreased more rapidly in transgenic mice infected by isolate 19504lux from France than in the mice infected by isolate LNP14354lux from Africa, suggesting significantly higher virulence for the serogroup X isolate from Africa (Figure 2).

We next focused on differences between CC181 isolates obtained since 2006 and those obtained in the 1990s. We detected 18 genes for which alleles were identical in all isolates obtained since 2006 and in LNP14354 (obtained in the 1990s) but differed in LNP13407 (obtained in the 1990s), and we detected 23 genes for which alleles were identical in all isolates obtained since 2006 and in LNP13407 but differed in LNP14354 (Table 2). 

**Table 2:** Identical loci in all *Neisseria meningitidis* isolates obtained since 2006 and in 1 isolate obtained in the 1990s (LNP14354 or LNP13407), sub-Saharan Africa and France, 1995–2008

All isolates obtained since 2006 and in LNP14354, n = 18 (gene)	All isolates obtained since 2006 and in LNP13407, n = 23 (gene)
16S_ribosomal DNA	NEIS0047 (*rfbB*)
NEIS0056 (*ctrB*)	NEIS0048 (*galE*)
NEIS0057 (*ctrC*)	NEIS0066 (*ctrE*)
NEIS0187	NEIS0092
NEIS0235	NEIS0095
NEIS0291 (*lot*)	NEIS0107
NEIS0303	NEIS0108
NEIS0342	NEIS0224
NEIS0620 (*maeA*)	NEIS0352
NEIS0665	NEIS0378
NEIS0695	NEIS0554
NEIS1034	NEIS0689
NEIS1558	NEIS0691
NEIS1651	NEIS0795
NEIS2083 (*mafA_MGI-3*)	NEIS1300
NEIS2474	NEIS1357
NEIS2484	NEIS1594
fHbp_PEPTIDEfrag_Pasteur	NEIS1706
	NEIS1777
	NEIS1978
	NEIS1986 (*lpt3*)
	NEIS2149
	NEIS2486

Most of these 41 genes encode hypothetical proteins; several encode proteins that are involved in meningococcal survival in the blood (Table 2; Figure 1, panel C). In particular, genes of interest were *fHbp* (encodes factor H binding protein), *lpt3* (encodes the phosphoethanolamine transferase), and *lot3* (encodes the lipooligosaccharides, O-acetyltransferase). All serogroup X isolates obtained since the outbreak of 2006 (including those from the BIGSdb platform) harbored the *lpt3* allele 45, which differs from allele 136 by the deletion of 2 codons (encoding Glu-211 and Ser-222 residues). These isolates do not react with the monoclonal antibody that recognizes immunotype L3,7,9 on the lipooligosaccharide (*15*). Replacing allele 45 with allele 136 by transformation enabled re-recognition of the epitope L3,7,9 (data not shown). The epitope L3,7,9 occurs frequently among invasive isolates, and antibodies against this epitope seem to be of importance for protection. Adding phosphoethanolamine on lipooligosaccharide may hide the epitope. This lipooligosaccharide modification may have enabled serogroup X to escape population immunity against lipooligosaccharides and contributed to its reemergence (*15*).

## Conclusions

The extensive characterization of *N. meningitidis* serogroup X isolates in the meningitis belt of Africa supports the emergence of virulent serogroup X isolates and the belief that CC181 may be considered a hyperinvasive genetic lineage of meningococci. The occurrence of the periodic epidemic waves may be caused by the emergence and spread of successive new clones of meningococci with enhanced virulence and an ability to escape population immunity. Our data underline the need for whole-genome sequencing for reliable tracking of meningococcal isolates and emphasizes the need for an effective vaccine against serogroup X (*5*).
